# Passing Events and Topics

**Published:** 1921-01-15

**Authors:** 


### Passing Events and Topics.

### The King's Concert to Patients.

The annual concert given by the King to patients in the Great Northern Central Hospital was held on January 4. A telegram of thanks from the patients was sent to His Majesty at Sandringham.

### No Registration for Belgian Practitioners.

As Belgium no longer affords to British medical practitioners the privileges of practising in Belgium, the Order in Council of January 7, 1915, giving recognition here to Belgian qualifications has been revoked, and persons possessing Belgian qualifications are no longer entitled to registration in this country. This does not, however, apply to any persons whose names have already been inscribed on the Register.

### A Great Charity Matinee.

Sir Oswald Stoll has placed the Coliseum at the disposal of the National Hospital for the Paralysed and Epileptic for the purpose of a matinée on February 22, as a slight recognition of the ungrudging and voluntary work which has been given by one of its medical staff, Dr. T. Grainger Stewart, M.D., in treating the disabled sailors and soldiers resident at the War Seal Foundation Home at Fulham. This welcome announcement was made at a large meeting of ladies, presided over by Princess Alice, Countess of Athlone, which was held on January 4 at the National Hospital, Queen Square.

### Post-Graduate Course on Infant and Child Welfare.

A course of twelve practical demonstrations on the "Management and Feeding of Infants and Young Children" will be given at the St. Marylebone General Dispensary by Dr. Eric Pritchard to qualified practitioners on Tuesdays and Thursdays in each week, beginning Tuesday, January 18, and ending Thursday, February 24. The Tuesday lectures will be at 10.30 a.m., and the Thursday lectures at 3 p.m. The course includes visits to the Nursery Training School, 1 Wellgarth Road, Golders Green. Tickets, price two guineas, are to be obtained from the Secretary of the Dispensary, 77 Welbeck Street, W. 1.

### Chelsea Hospital for Women.

An extraordinary record was created at the Chelsea Hospital for Women in the first half of 1920. The major operations numbered 259, the minor 156, and there was only one death. Over the whole year the rate of mortality on all cases was under 1¼ per cent., which cannot be far from a record for twelve months' work of a similar nature. The Convalescent Home at St. Leonards-on-Sea has been of inestimable service. £1,800 is needed for the Nurses' Home, and a dinner to raise this amount is to be held in the spring, under the joint presidency of the Marquess of Londonderry, President of the hospital, and the Countess of Ilchester, President of the Ladies' Committee.

### Miss Miller's Bible Classes.

A real welcome awaits any nurse friends of Miss A. E. Miller (late Secretary of the Y.W.C.A. Central Institute) who care to attend the Bible Classes for men and women which she holds in the Fyvie Hall of the Polytechnic Institute, Regent Street, every Thursday, from 7.45 to 8.45 p.m. The studies are illustrated by blackboard diagrams.

### Warneford Hospital Ball, Leamington.

For the second time since the war the Warneford Hospital Ball took place on January 5 in the Leamington Town Hall. About 300 people were present, and the ball was a great success. The floral and other decorations were particularly beautiful, and revived memories of pre-war splendours.

### The Nurses' Dance.

Having decided to give a dance and entertainment in appreciation of the services of the nurses and staff at the Infectious Diseases Hospitals and Sanatorium, the Hull Corporation Health Committee recommends an expenditure of from £75 to £80 for the interesting affair. It is proposed to invite fifty-five nurses, each of whom will be able to bring a friend. A supper is to be given as well.

### Memorial to Jack Cornwall.

In order to provide a number of cottage homes for the use of disabled sailors—which, it is suggested, shall form a memorial to the late Jack Cornwall—a site for the purpose has been offered in East Ham by Sir John Bethel, M.P.

